# Impact of the fermentation broth of *Ganoderma lucidum* on the quality of Chinese steamed bread

**DOI:** 10.1186/s13568-019-0859-5

**Published:** 2019-08-27

**Authors:** Zhao Guowei, Wei Lili, Liu Yufeng, Wang Hailei

**Affiliations:** 1 College of Life Sciences, Henan Normal University, Xinxiang, 453007 China; 2 Department of Life Science and Engineering, Jining College, Qufu, 273155 China

**Keywords:** Laccase, Amylose, Amylase, Chinese steamed bread, *Ganoderma lucidum*, Amylopectin

## Abstract

The potential of fermentation broth of *Ganoderma lucidum* (FB_G_) in improving the quality of Chinese steamed bread (CSB) was firstly evaluated. The sensory quality scores of CSB treated by FB_G_ are significantly higher than that of CSB in the control, and texture profile analysis also indicates the increase of CSB hardness and chewiness caused by FB_G_. Observation on micro-structure of CSB shows that formation of larger pores and expansion of starch granules are the important reasons for the improvement of CSB specific volume (vol_S_), and granule expansion is due to that gluten network distributed in CSB is destroyed as a result of cross-linkage of flour proteins catalyzed by laccase, which makes starch granules releasing from the network easily contact with steam or other enzymes during the proofing and steaming of dough. Moreover, FB_G_ contains amylases which not only convert amylopectin to amylose, but also degrade starch to glucose, maltose and polysaccharides, correspondingly resulting in changes of amylose/amylopectin (Ae/An) ratio of flour and CSB vol_S_, and the latter is because more CO_2_ produced by the yeast during CSB making leads to the larger pore area in crumb. Both hardness and chewiness are determined by the comprehensive effect of protein cross-linkage, Ae/An ratio and vol_S_ change, and this viewpoint gives a logical explanation for the effects of 0.025–0.10 ml/g of FB_G_ on hardness and chewiness of CSB.

## Introduction

Chinese steamed bread (CSB) is a fermented wheat flour product. Its preparation process is similar to that of western-style pan bread, but the final product is steamed in a steamer, not baked in an oven. Steaming process has an advantage over baking since it uses water vapor temperature which is much lower than baking temperature (around 180–220 °C). Therefore, nutrients might be better retained when compared to the baked bread (Victoria et al. 2013). Over the centuries, within the intercommunication of food culture among different countries, CSB has spread from China to other Asian, North America and European countries (Wu et al. 2012). In CSB making process, although protein and starch, as the main components in flour, were the most important factors in determining CSB quality, lipids, non-starch polysaccharides and especially some enzymes also played a comparatively important role (Oliveira et al. 2014; Singh et al. 2010). The effects of several enzymes including laccase, xylanase and tyrosinase on the properties of oat and wheat dough were investigated extensively (Flander et al. 2011; Selinheimo et al. 2006; Su et al. 2005), but, the higher cost of these purified enzymes becomes a bottleneck impeding their commercial application in view of the low added value of CSB as a daily consumer goods.

*Ganoderma lucidum* is a medicinal mushroom that has been used as a home remedy for the general promotion of health and longevity in East Asia, and it is popularly used worldwide in the form of dietary supplements (Stanley et al. 2005). The information indicates that *G. lucidum* could be potentially acceptable for food applications. The fermentation broth of *G. lucidum* (FB_G_) obtained by submerged cultivation is a traditional Chinese medicine which is used to treat chronic tracheitis and hypercholesterolemia in China, and it contains many useful second metabolites including a series of enzymes, polysaccharides and triterpenoids that are considered to possess multiple biological activities (Li et al. 2011; Xu et al. 2010; Zhou et al. 2014).

Taking these considerations into account, FB_G_ was prepared and added to wheat flour as a relatively inexpensive food additive. The aims of this study are (i) to investigate potential of FB_G_ in improving CSB quality, and (ii) to study impact of FB_G_ on CSB properties and elucidate its function during CSB making. To the best of our knowledge, there is little information available in the previous literature about application of FB_G_ to CSB making.

## Materials and methods

### Chemicals, raw materials and strains

The standard samples of amylopectin and amylose, guaiacol and 2, 2-azino-bis (3-ethylbenzthiazoline-6-sulfonic acid) diammonium salt (ABTS) were obtained from Sigma. Wheat flour containing 70.7% of starch, 12.1% of moisture, 10.4% of protein and 0.53% of ash was supplied by Zhengzhou Jingyuan Flour Co., Ltd., China. The dehydrated yeast (*Saccharomyces cerevisiae*) bought from Angel Yeast Co., Ltd., China, was a kind of commercial instant yeast for common production of CSB. *G. lucidu*m (CGMCC 5.533) was obtained from China General Microbiological Culture Collection Center, Beijing, China, and the fungus was maintained on potato dextrose agar (PDA) slant at 4 °C and sub-cultured every 3 months.

### Preparation of the FB_G_

*Ganoderma lucidum* was grown on PDA for 3 d at 28 °C, and mycelial suspension was prepared using the sterile water. During the submerged cultivation, the Erlenmeyer flasks (500 ml) containing 100 ml of liquid medium were inoculated with 5 ml of mycelial suspension, and incubated in a thermostat shaker at 28 °C and 180 rpm. The liquid medium was composed of 40 g/l glucose, 20 g/l wheat bran, 10 g/l peptone, 3.0 g/l KH_2_PO_4_ and 1.5 g/l MgSO_4_·7H_2_O. After 7-day cultivation, the medium was centrifuged at 5000 rpm for 10 min and the supernatant was defined as FB_G_.

### Zymogram analysis of laccase

During the submerged cultivation, laccase activity was determined with ABTS as a substrate (Zilly et al. 2012). After the cultivation, zymogram of laccase in FB_G_ was analyzed by native polyacrylamide gel electrophoresis (native-PAGE) which was performed under non-denaturing conditions. The separating and stacking gels contained 12 and 5% concentrations of acrylamide, respectively. The buffer solutions were 50 mM Tris–HCl (pH 9.5) for the separating gel and 18 mM Tris–HCl (pH 7.5) for the stacking gel. The electrode reservoir solution was 25 mM Tris and 190 mM glycine (pH 8.4). After electrophoresis, visualization of protein bands was achieved by Coomassie brilliant blue (R350, Pharmacia) staining. Activity staining of laccase was performed by incubating the PAGE gel in 0.5 mM sodium acetate buffer (pH 5.5) containing 0.02% guaiacol at 25 ± 1 °C.

### Making process and quality test of CSB

The making process of CSB is as follows: in the treatment group, FB_G_ was added to flour at the levels of 0.025, 0.05 and 0.10 ml/g, respectively; in the control group, no FB_G_ was added. The formulation of CSB is: wheat flour 100.0 g, dehydrated yeast 1.0 g and distilled water (50 ml). The mixture of flour and yeast was kneaded to form dough, and the dough was sheeted for 20 times and divided into several pieces. The piece doughs were rounded, molded manually and proofed for 50 min at 38 °C and 85% relative humidity. The proofed doughs were steamed for 20 min in a steamer.

The quality of CSB was evaluated according to the evaluation criteria of CSB (SB/T10139-93, Ministry of Internal Trade, China). Volume was measured by rapeseed displacement method (Pyler 1988) after the cooling. The pore in crumb was photographed and observed after slicing from the middle using a blade (Leica 818, Germany). Sensory evaluation was judged by thirty experts according to the 100-point evaluation scheme described in Table 1 (Lin et al. 2012).

**Table 1:** Sensory evaluation criteria and scores of CSBs treated by FB_G_ with the different dosages

CSB attributes	Max. score	Evaluation criteria	FB_G_ dosage (ml/g)			
			0	0.025	0.05	0.10
Specific volume (SV)	20	20-(2.3-SV)/0.1	17.1 ± 0.2	18.1 ± 0.1	18.9 ± 0.2	19.2 ± 0.2
External	15	Good symmetry, smooth skin: 12.1–15.0; medium: 9.1–12.0; bad symmetry, coarse skin: 1.0–9.0	12.0 ± 0.08	13.0 ± 0.09	13.0 ± 0.07	13.0 ± 0.05
Crumb’s structure	15	Regular and small crumb’s pore size: 12.1–15.0; medium: 9.1–12.0; irregular and large crumb’s pore size: 1.0–9.0	8.0 ± 0.07	14.0 ± 0.10	7.0 ± 0.06	7.1 ± 0.10
Recovery after compression	20	Good recovery, firm bite: 16.1–20.0; medium: 12.1–16.0; bad recovery, soft bite: 1.0–12.0	13.9 ± 0.10	14.5 ± 0.10	17.0 ± 0.20	14.0 ± 0.30
Stickiness	15	Chewier: 12.1–15.0; medium: 9.1–12.0; sticky: 1.0–9.0	10.0 ± 0.21	11.6 ± 0.10	12.5 ± 0.20	11.0 ± 0.25
Color	10	White, milky: 8.1–10.0; medium: 6.1–8.0; dark: 1.0–6.0	8.7 ± 0.10	8.1 ± 0.06	7.9 ± 0.10	7.5 ± 0.07
Flavor and smell	5	Natural wheat flavor, no bad odour: 4.1–5.0; medium: 3.1–4.0; bad odour: 1.0–3.0	4.1 ± 0.04	4.9 ± 0.05	4.8 ± 0.05	4.9 ± 0.06
Total score	100		73.3 ± 0.51	90.1 ± 0.5	078.1 ± 0.42	80.1 ± 0.37

### Texture profile analysis (TPA)

TPA was determined by a TA-XT2i texture analyser (Stable Micro Systems, Ltd., Godalming, UK) according to the method described by Kadan et al. (2001). CSB was sliced horizontally and a piece, 20 mm height, was compressed to 30% of its height. The test conditions were as follows: pre-test speed 3 mm/s, test speed 1 mm/s, post-test speed 5 mm/s and trigger force 5 g. The parameters including hardness, springiness, chewiness and resilience were evaluated based on the data of TPA.

### Micro-structure observation

The CSB cubes (each side of the cube = 0.5 cm) prepared as described by Selinheimo et al. (2007) were cut into sections with the thickness of 15 µm by a Leica HM355 rotary microtome (Germany), and then transferred onto glass slides. The sections were stained with Light green solution (0.1%) and Lugol’s iodine solution (I_2_, 0.33%, w/v; KI, 0.67%, w/v) to dye starch to dark or brown and protein to green, respectively. The stained samples were washed with distilled water for 1 min, and then observed under a microscope with an image analysis system (Image-Pro Plus, V4.0, Media Cybernetics).

### Protein cross-linkage catalyzed by laccase

Laccase in FB_G_ was purified by two (NH_4_)_2_SO_4_ precipitation steps: (i) 40% saturated (NH_4_)_2_SO_4_ solution was used to remove other proteins; and (ii) 70% saturated (NH_4_)_2_SO_4_ solution was used to precipitate laccase. The resulting precipitate was dialyzed to remove (NH_4_)_2_SO_4_, and the desalted enzyme solution was applied to a DEAE52 column pre-equilibrated with sodium phosphate buffer (pH 7.2). The absorbed laccase was eluted using 0.2 M NaCl, and the active fractions were collected, dialyzed, and concentrated by lyophilization.

Protein cross-linkage assay was conducted according to the following steps (Gao et al. 2010): after addition of the purified laccase (0.5 and 2.0 ml/g, respectively), the flour was kneaded into dough, and proofed for 50 min at 38 °C. Starch in the dough was removed by washing using the sterile water, and fat was extracted twice with 100 ml acetone. The proteins left were dissolved in 0.1 M sodium phosphate buffer (pH 6.5), and the protein solution (1.5 mg/l) was analyzed by sodium dodecyl sulfate polyacrylamide gel electrophoresis (SDS-PAGE).

### Starch degradation catalyzed by FB_G_

Two tests were conducted to study the impact of FB_G_ on starch. In test 1, FB_G_ (0.025 0.05 and 0.10 ml/g) was added to three tubes each containing 30 ml of amylopectin solution (60 mg/l). After 3 h reaction at 25 °C and pH 5.5, color change of solution was recorded and contents of both amylopectin and amylose were determined by the method of Adejumo et al. (2013). In test 2, FB_G_ (0.10 ml/g) was added to amylopectin (0.67 g/ml) and amylose (0.83 g/ml) suspension, respectively. After 3 h reaction at 25 °C and pH 5.5, the supernatants were analyzed by silica gel thin layer chromatograph (TLC) using n-butanol: ethyl acetate: pyridine: water (6:1:5:4) as mobile phase. Both glucose and maltose were measured by the high-performance liquid chromatography (HPLC) with refractive index detection (Liu et al. 2006).

### Effect of glucose and maltose on CSB characteristics

Four treatments (T1–T4) were designed. In T1, no glucose and maltose was added to flour; in T2–T4, both glucose and maltose (24.2 mg/g glucose + 1.7 mg/l maltose; 47.4 mg/g glucose + 3.0 mg/l maltose; 69.9 mg/g glucose + 4.1 mg/l maltose) were added to flour, respectively. All of the flours were used to make CSBs, and the CSBs obtained were sliced from the middle. The pore number and total pore area in CSB slices were analyzed quantitatively by digital image analysis (Sapirstein et al. 1994; Zghal et al. 2001) using the software of Matlab 7.0 (Mathworks, Natick, MA, USA).

### Statistical methods

The obtained data in this work were presented as mean ± standard deviation of multiple measurements, and the statistical analysis of data was carried out using the software SPSS 12.0 for Windows. One-way ANOVA was used to analyze the data to ascertain whether the change of Ae/An ratio significantly affected CSB properties. The level of significance of correlation coefficient was analyzed by a two-tailed test.

## Results

### Submerged cultivation and zymogram analysis of laccase

**Fig. 1:**
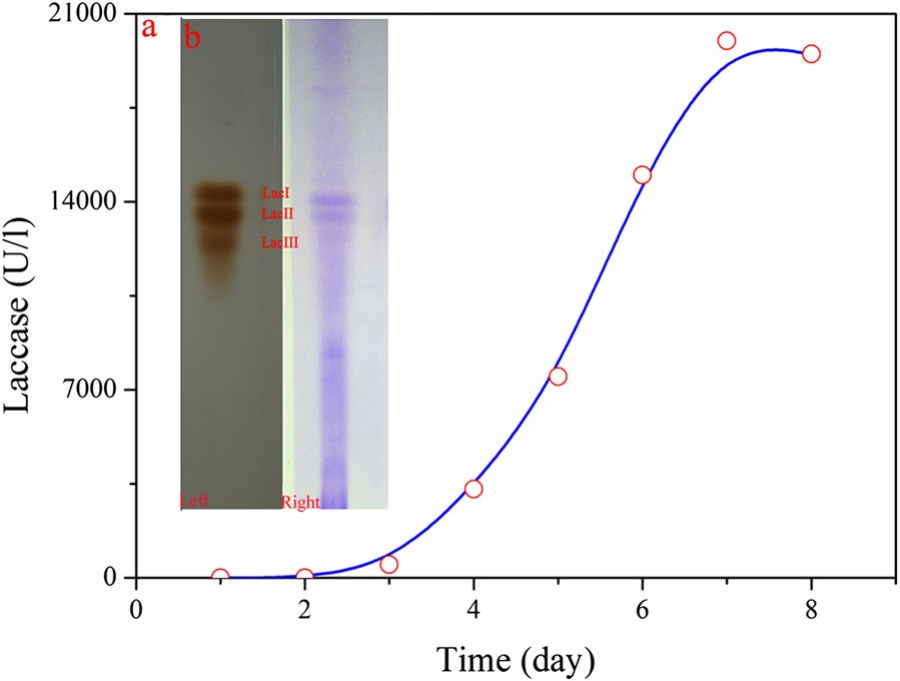
Laccase activity measurement and isoenzyme analysis. **a** Laccase activity varied with time during the submerged fermentation; **b** native-PAGE analysis of laccase isoenzymes: left, guaiacol staining; right, Coomassie brilliant blue staining

The *G. lucidum* used in this work is a good laccase producer (Wang et al. 2013), and thus laccase activity was measured during the submerged cultivation. Laccase appeared in FB_G_ on day 2, and then its activity increased with time. On day 7, a maximum laccase activity, 18,000 U/l, was obtained at 500 ml shake-flask level (Fig. 1a). The FB_G_ obtained was analyzed by native-PAGE, and three clear and distinct bands were observed after incubating the gel with the laccase substrate, guaiacol (Fig. 1b-left), which indicates the presence of three laccase isoforms (Lac I–III) in FB_G_ (Ko et al. 2001). Native-PAGE gel stained with Coomassie brilliant blue shows that both Lac I and Lac II are the main laccase isoforms, and they are much higher than Lac III in protein content (Fig. 1b-right).

### Sensory evaluation

CSB quality assessment is largely based on personal judgment and subjective qualitative evaluation, and the results reflect the consumer preferences (Shah et al. 2006). Sensory evaluation analysis clearly demonstrates that FB_G_ has a positive effect on CSB quality. It remarkably modifies organoleptic properties of CSB including the specific volume (vol_S_), external, crumb’s structure, recovery after compression, stickiness, color, flavor and smell. Compared with the control, appearance of FB_G_ in flour (0.025–0.10 ml/g) improved the sensory evaluation score of CSB. CSB supplementing 0.025 ml/g of FB_G_ fetched the highest total score (Table 1), and it was significantly superior over the other CSBs with respect to attributes except color. Color of all of the treated CSBs became a little darker with the increase of FB_G_ dosage due to the fact that laccase catalyzes amino acids or phenolic acids such as ferulic acid in flour to produce some color substances (Selinheimo et al. 2007).

### Specific volume

**Fig. 2:**
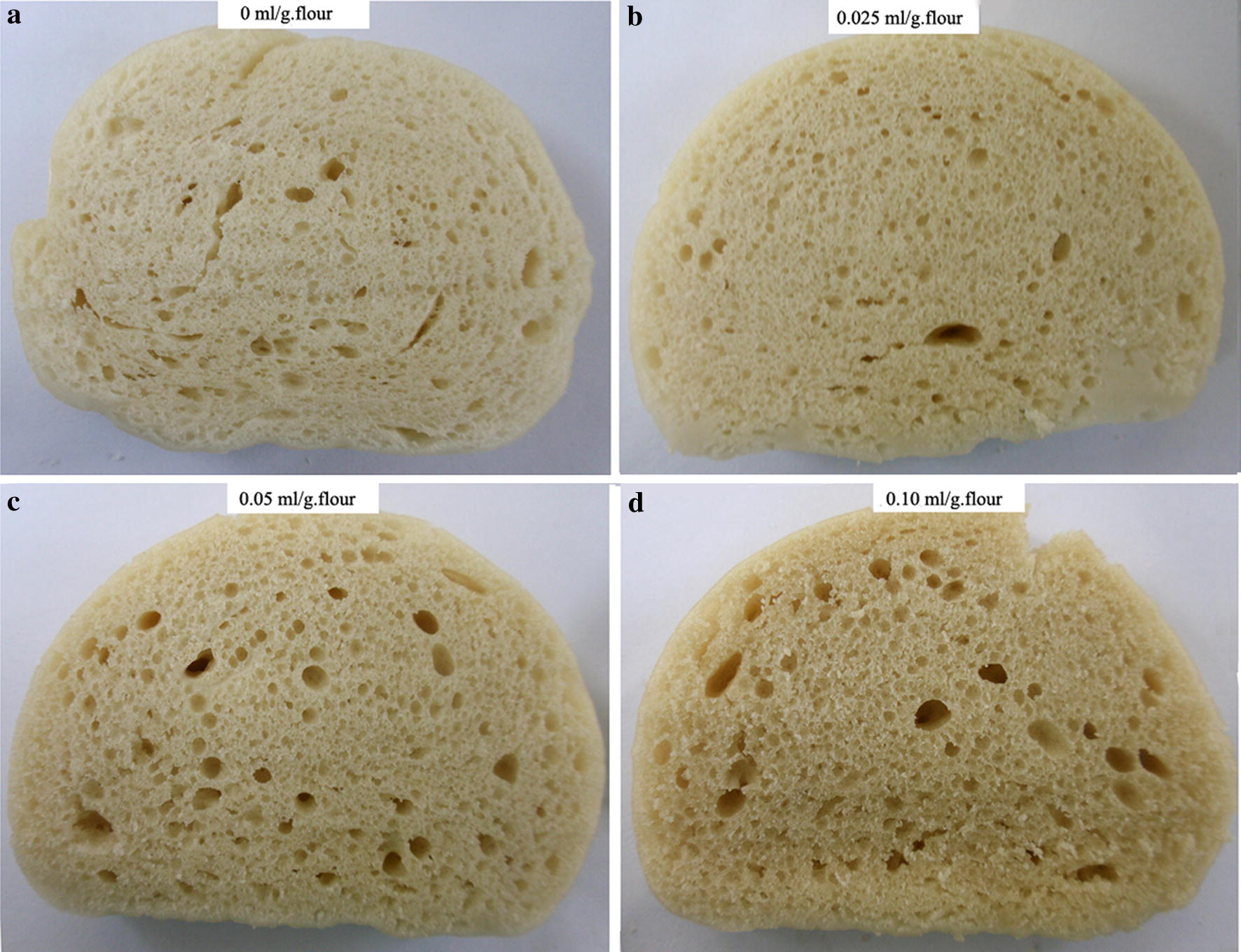
Visual examination of the effect of FB_G_ on pore size of the crumbs. **a** The reference; **b** 0.025 ml/g; **c** 0.05 ml/g; **d** 0.10 ml/g

Volume is the most important quality parameter for CSB. Vol_S_ of the treated CSBs increased with the FB_G_ dosage, and all of them are larger than that of the control. Thus, it is feasible to increase CSB volume by FB_G_. Photographs of CSB slices (Fig. 2) show that the pore size in CSB treated with 0.025 ml/g of FB_G_ was noticeable regular when compared to that of the control. The higher FB_G_ dosage (≥ 0.05 ml/g) changed pore size in CSB to more irregular and larger, and this phenomenon can explain the scores in crumb’s structure of CSBs in sensory evaluation. Obviously, formation of the large pore is also an important reason for the larger vol_S_ of CSBs treated with FB_G_.

### Effect of FB_G_ on size distribution of starch granules

**Fig. 3:**
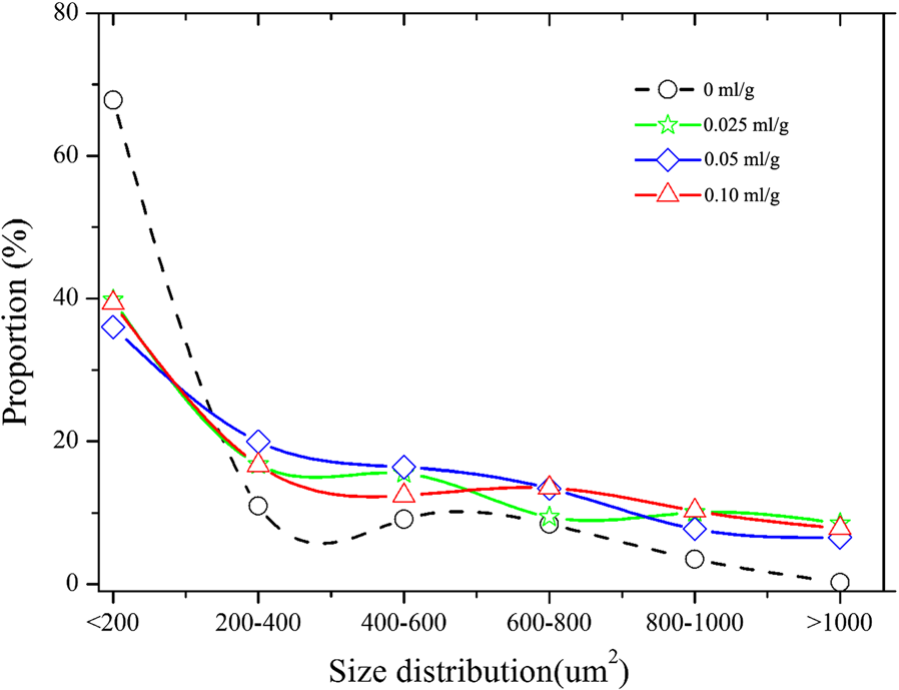
Size distribution of starch granules in CSBs

A more interesting phenomenon was found by observing CSB micro-structure: the size of starch granules changes with the FB_G_ dosage. Compared with the treatment group, number of the small starch granules (< 200 μm^2^) in the control group decreased, while number of the larger starch granules (> 200 μm^2^) significantly increased (Fig. 3). In view of the stable amount of total starch granules, the expansion of starch granule will also lead to the larger vol_S_ of CSB. Thus, we can also explain enlargement of CSB vol_S_ from the perspective of size change of starch granules.

**Fig. 4:**
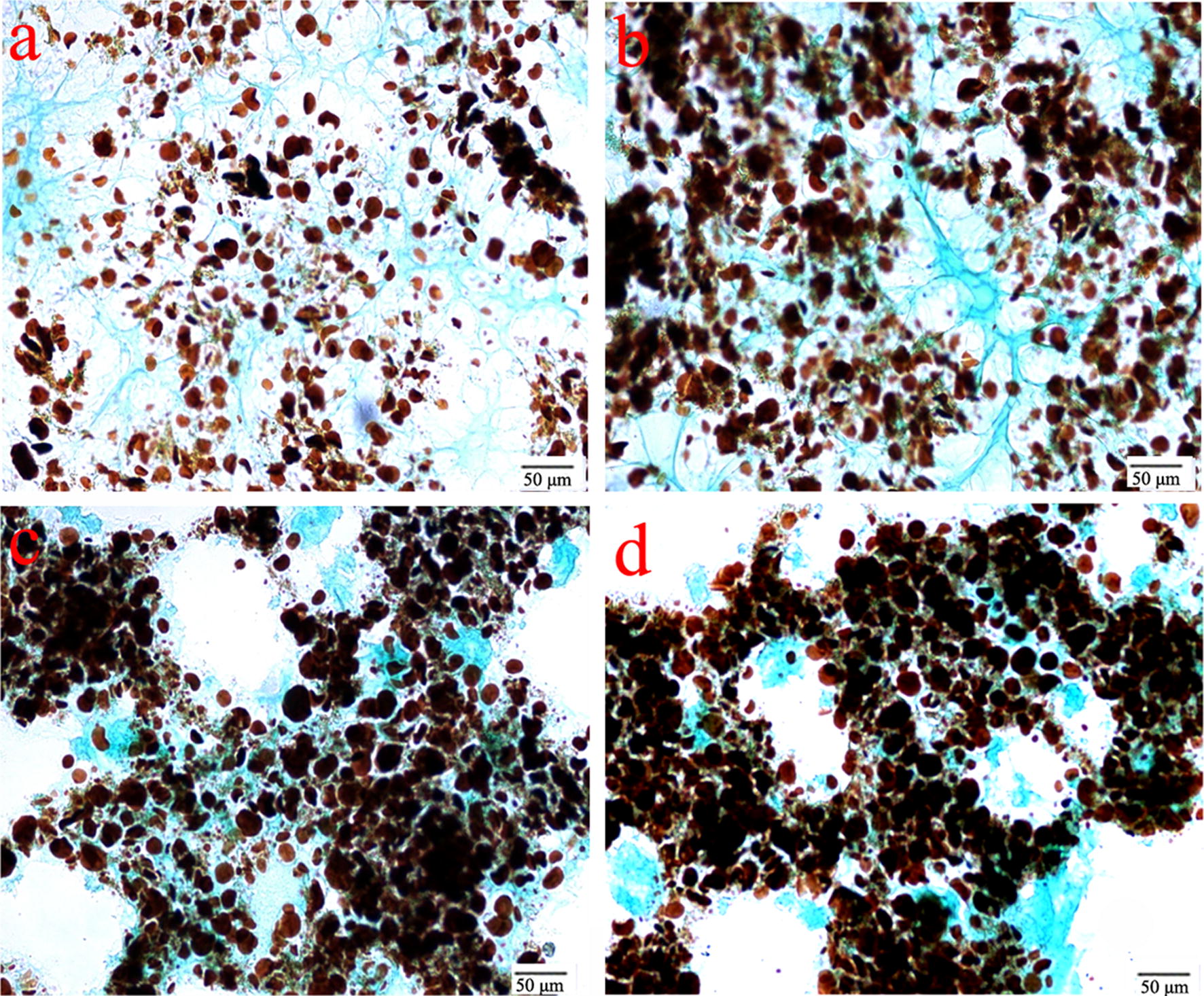
Microscopy examination of the effect of FB_G_ on proteins and starch granules in the crumbs. **a** The reference; **b** 0.025 ml/g; **c** 0.05 ml/g; **d** 0.10 ml/g

Considering that CSBs in both the control and treatment group were performed the same processing except the FB_G_ dosage, the appearance of larger starch granules should be attributed to the addition of FB_G_. Therefore, the effect of FB_G_ on flour proteins was studied. After the staining, the yellow or dark starch granules in the control were entrapped in the network of gluten protein (Fig. 4a). However, the appearance of 0.025 ml/g of FB_G_ led to a clear cross-linkage of proteins (Fig. 4b). When FB_G_ was increased to 0.05 and 0.10 ml/g, the large protein-rich areas were formed (Fig. 4c, d), and the uniform protein network suffered serious devastation.

Laccase activity in FB_G_ was approximately 20 U/ml, and the fungal laccases are able to cross-link proteins (Ercili et al. 2009; Figueroa-Espinoza and Rouau 1998; Figueroa-Espinoza et al. 1999). Thus, laccase in FB_G_ was purified by (NH_4_)_2_SO_4_ precipitation and DEAE52 column chromatography. After the two-step purification, the specific activity of laccase was increased from 1.66 to 17.60 U/mg (Table 2). The enzyme was purified to 10.6 fold, and the recovery rate was 38%.

**Table 2:** Summary of laccase purification

Purification	Volume (ml)	Laccase activity (U)	Protein (mg)	Specific activity (U/mg)	Purification multiple	Recovery rate (%)
FB_G_	10	501	302.7	1.66	1.00	100
(NH_4_)_2_SO_4_ precipitation	–	395	103.2	3.80	2.31	79
DEAE52 column chromatography	8	190	10.8	17.6	4.60	38

**Fig. 5:**
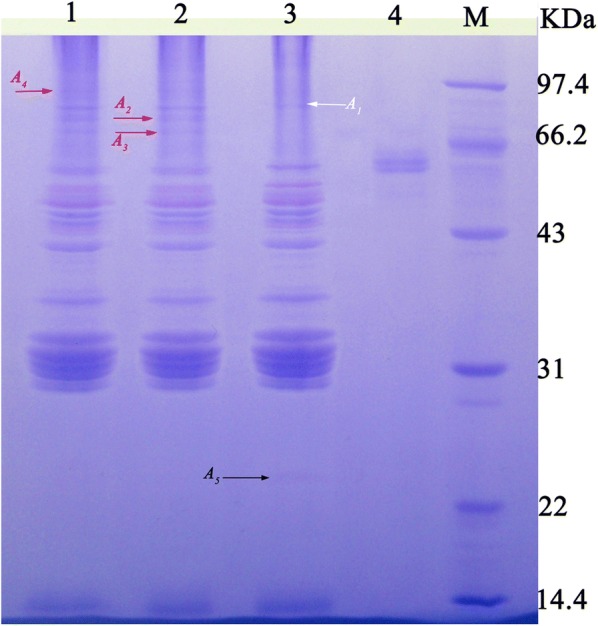
SDS-PAGE analysis on the cross-linkage of flour proteins. Lanes: (1) flour treated with 2.0 U/g of laccase; (2) flour treated with 0.5 U/g of laccase; (3) the reference; (4) laccase; (M) the molecular weight marker. Major changes are illustrated with arrows (A_1_–A_5_)

Ability of the purification laccase to cross-link proteins was determined. Figure 5 clearly shows that 0.5 and 2.0 U/g of laccase can cross-link flour proteins by SDS-PAGE analysis. Compared with the reference, the content of protein (*A*_1_) with the higher molecular weight was increased, and also some new proteins (*A*_2–_*A*_4_) appeared in the gel (Fig. 5, lanes 1–2). In addition, a lower molecular weight protein (*A*_5_) presenting in lane 3 disappeared in lanes 1–2. A probable explanation is that the protein was used to form the higher molecular weight products by cross-linking with other substances. Thus, the devastation of uniform protein network in CSB might be attributed to cross-linkage of proteins catalyzed by laccase (Flander et al. 2008; Labat et al. 2000).

### TPA of CSB

**Fig. 6:**
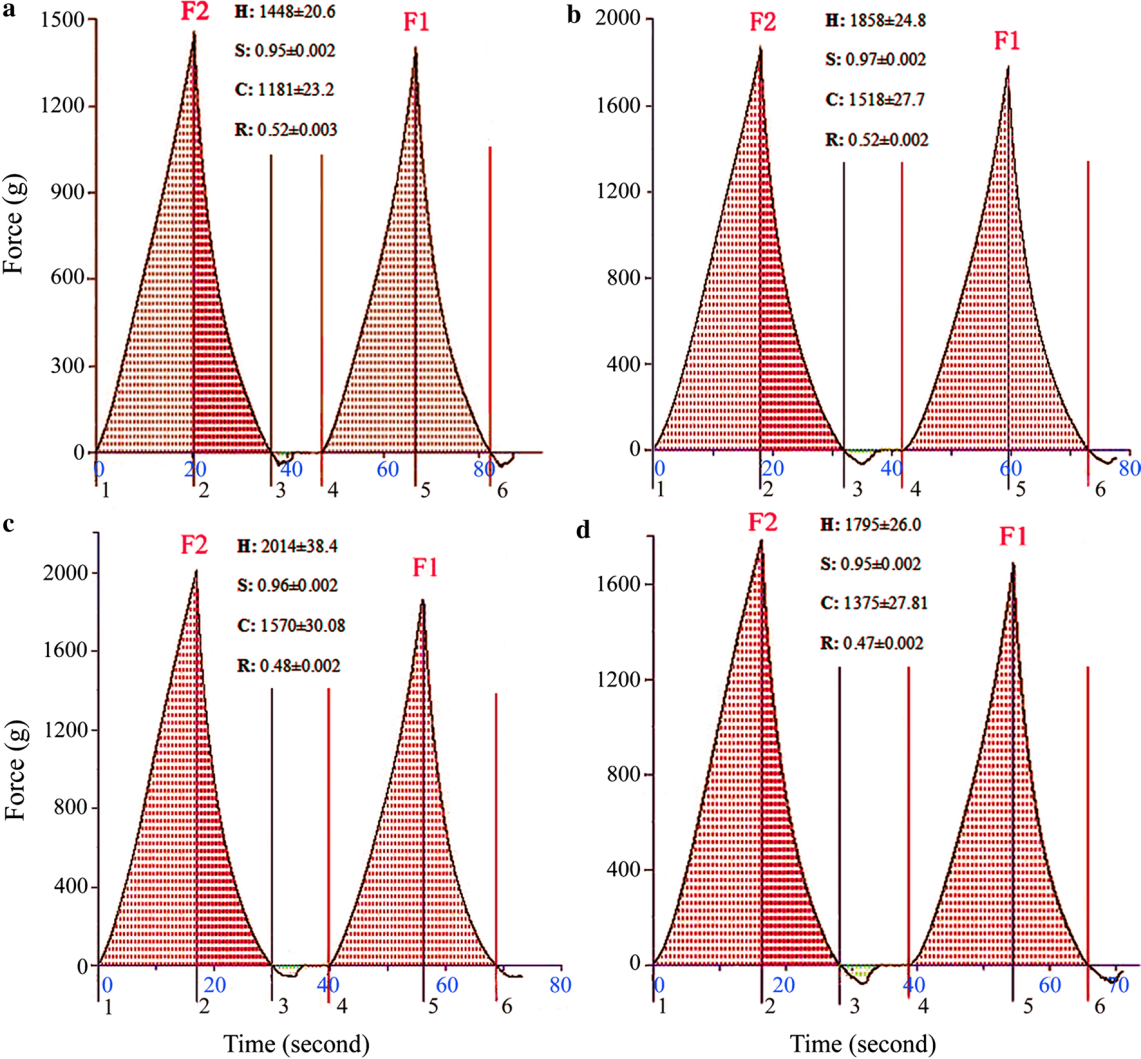
Texture profile analysis of CSBs treated by FB_G_. **a** The reference; **b** 0.025 ml/g; **c** 0.05 ml/g; **d** 0.10 ml/g. Interpretation of texture parameters: Hardness = F2; Springiness = time_4–5_/time_1–2_; Chewiness = F2 × (area_4–6_/area_1–3_) × (time_4–5_/time_1–2_); Resilience = area_2–3_/area_1–2_

As shown in Table 1, FB_G_ at the dosages of 0.025–0.10 ml/g gives CSB a better score in recovery after compression and stickiness in sensory evaluation. Since the scores of recovery after compression and stickiness correspond to hardness and chewiness of CSB in TPA, respectively. Thus, TPA was conducted to validate the reliability of sensory evaluation. As shown in Fig. 6, the addition of 0.025–0.10 ml/g of FB_G_ in flour increased CSB hardness, and the maximum hardness, 2014, was obtained at 0.05 ml/g of FB_G_. However, the hardness decreased at the FB_G_ dosage of 0.10 ml/g. The same tendency in chewiness was observed. In addition, FB_G_ had no significant influence on resilience and springiness of CSB. Hence, the sensory evaluation of CSB attributes is supported by TPA data.

## Discussion

### Effect of cross-linkage of protein on CSB characteristics

The facts above mentioned demonstrate that 0.025–0.10 ml/g of FB_G_ had a significant influence on CSB hardness and chewiness. The previous literature has proven that laccase is able to improve CSB hardness by cross-linking proteinaceous food matrices (Selinheimo et al. 2006; Minussi et al. 2002), and in this work, both micro-structure observation and SDS-PAGE analysis show the ability of laccase in FB_G_ to cross-link flour proteins. Thus, cross-linkage of protein catalyzed by laccase is one of the important reasons for improvement of CSB hardness and chewiness. In addition, the cross-linkage of protein also can explain formation of the larger starch granules during CSB making: the cross-linkage causes the destruction of gluten network, which results in the release of small starch granules embedded in the network. The small granules easily contact with steam or enzymes during the proofing and steaming of dough, leading to the granule expansion such as the expansion of amylopectin accounting approximately for 65–81% of starch (Nakamura 2002), and then formation of the larger starch granules. Nutritionally, the small starch granules embedded in the protein network are dense, and uneasy to be hydrolyzed, while the larger starch granules are suitable to be digested because of their loose structure.

### Effect of amylopectin conversion on CSB characteristics

**Fig. 7:**
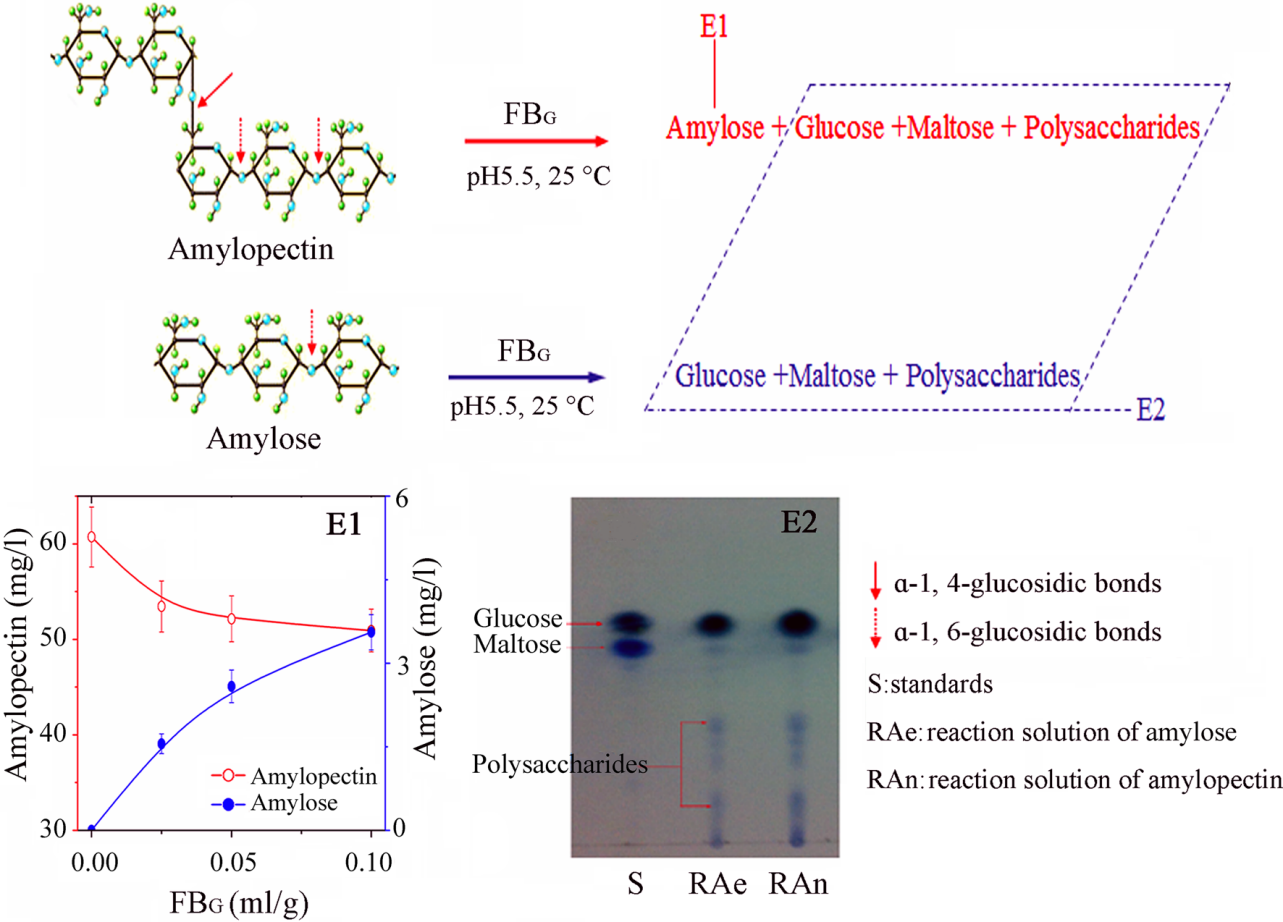
Reactions and evidences of starch degradation catalyzed by FB_G_. E1, The concentrations of amylopectin and amylose after 3 h reaction; E2, TLC analysis of the products of amylopectin and amylose treated by FB_G_

The main component of flour, starch, plays an important role in determining CSB quality. Therefore, the effect of FB_G_ on starch was studied. Amylopectin and amylose are the main components of starch in wheat flour (Zou et al. 2012), and they are stained purple or amaranth and brown or blue by I_2_, respectively. In test 1, after addition of FB_G_ to the tubes, the solutions became browner with the increase of FB_G_ dosage, which indicates the production of amylose (Fig. 7). The measurement data (Fig. 7-E1) also prove the appearance of amylose in the reaction system. Accompanying with the decrease of amylopectin, 1.55, 2.58 and 3.56 mg/l of amylose appeared in the tubes added 0.025, 0.05 and 0.10 ml/g of FB_G_, respectively. Therefore, FB_G_ should contain isoamylase which is able to convert amylopectin to amylose by breaking ɑ-1, 6-glucosidic bond (Kudanga et al. 2011; Zhu et al. 2013). To the best of our knowledge, this is the first report about the appearance of isoamylase during submerged cultivation of *G. lucidum*.

**Fig. 8:**
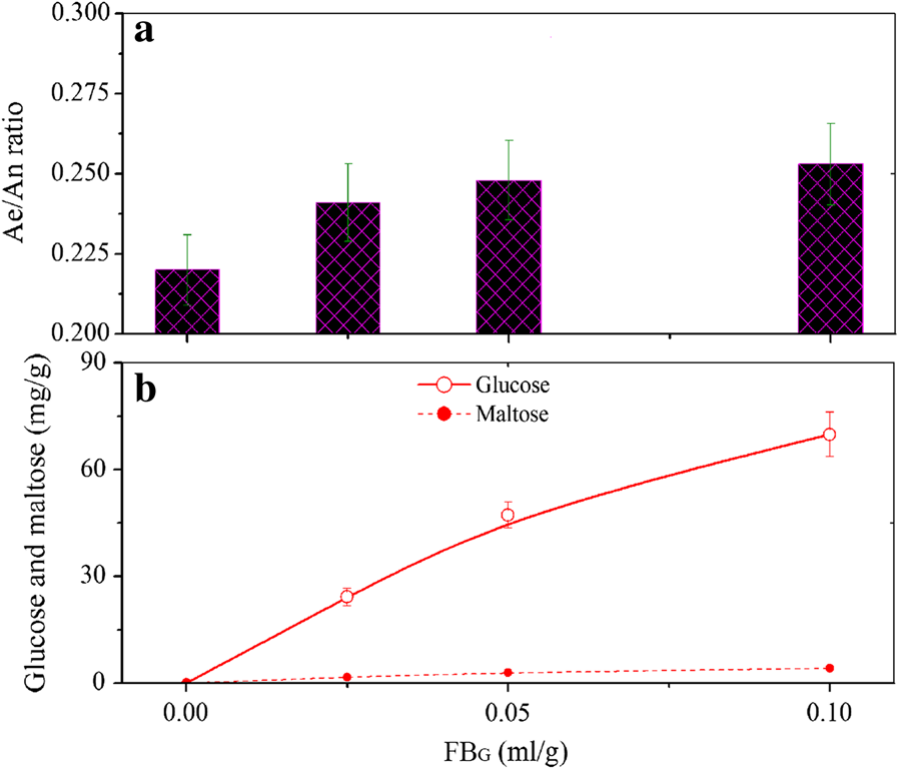
Variations of Ae/An ratio as well as glucose and maltose content with the FB_G_ dosage. **a** Variation of Ae/An ratio; **b** variations of glucose and maltase content

During CSB making, the conversion from amylopectin to amylose means the change of amylose/amylopectin (Ae/An) ratio of flour. Figure 8a demonstrates that 0.025–0.10 ml/g of FB_G_ increases Ae/An ratio. Ae/An ratio of flour was adjusted by the commercial amylopectin and amylose in order to investigate its effect on CSB characteristics, and after the adjustment, the flour with the different Ae/An ratio was used to make CSB. The results show that Ae/An ratio is positively correlative with hardness and chewiness of CSB (Table 3). Two-tailed test indicates that correlation coefficients are 0.972 and 0.963, respectively, and correlations are significant at the 0.01 level. Thus, the enhancement of Ae/An ratio of flour induced by 0.025–0.10 ml/g of FB_G_ also contributes to improvement of hardness and chewiness of CSB.

**Table 3:** Effect of Ae/An ratio on the hardness and chewiness of CSB

Flour	Starch (%)	Ae (%)	An (%)	Ae/An	Hardness (g)	Chewiness (g)
1	67.5	13.1	54.4	0.241	1630	1410
2	70.0	12.8	57.2	0.224	1460	1210
3	70.4	15.1	55.3	0.273	2810	1910
4	69.1	14.5	58.5	0.248	1980	1620
5	65.5	13.4	52.1	0.257	2210	1830
6	71.3	14.4	56.9	0.253	2150	1740

An: Amylopectin; Ae: amylose

### Starch degradation

Now, both protein cross-linkage and change of Ae/An ratio catalyze by FB_G_ can be used to explain the improvement of hardness and chewiness of CSB. However, neither of them can explain why both hardness and chewiness decreased at the FB_G_ dosage of 0.10 ml/g. In Fig. 7-E1, it should be noted that only 5.9% amylopectin was converted to amylose when amylopectin solution was treated by 0.10 ml/g of FB_G_, and the other products include glucose, maltose and polysaccharides also were produced during the reaction (Fig. 7-E2). In addition, amylose is degraded by FB_G_ too. When amylose was treated by 0.10 ml/g of FB_G_ for 3 h, the products in supernatant include glucose, maltose and other soluble polysaccharides by TLC analysis. These evidences show that the enzymes in FB_G_ also act on ɑ-1, 4-glucosidic bonds of amylopectin and amylose. Based on the analysis of degradation products of starch, the FB_G_ used at least has ɑ-amylase activity, and this result accords with our previous report (Li et al. 2011).

### Effect of glucose and maltose on CSB characteristics

The facts above mentioned prove that the appearance of FB_G_ in flour leads to degradation of a small amount of starch. Figure 8b shows that approximately 24.2, 47.4 and 69.9 mg/g of glucose as well as 1.7, 3.0 and 4.1 mg/g of maltose were correspondingly produced when 0.025–0.10 ml/g of FB_G_ was added to flour. Analysis on the effect of glucose and maltose on CSB characteristics shows that CSB slice in T2 obtained the maximum pore number. However, the total pore area in T4 was the largest in the four treatments (Table 4). In addition, CSB vol_S_ increased with improvement of the total pore area, but the hardness and chewiness decreased with it. These results were in accordance with the description of Fig. 3. Thus, the addition of glucose and maltose can enlarge vol_S_ and reduce hardness and chewiness of CSB. The reason is that *S. cerevisiae* was added to flour during CSB making, and both glucose and maltose are carbon resources for the growth of *S. cerevisiae*. The appearance of glucose and maltose in flour makes the yeast produce more CO_2_ during the fermentation phase, resulting in the appearance of the larger pore area in crumb. The larger pore area, on one hand, leads to enlargement of CSB volume; on the other hand, it decreases hardness and chewiness since the gas in CSB is easy compressed. Similar results have been reported in the literature that addition of ɑ-amylase can increase CSB vol_S_ by increasing gas production at the fermentation stage (Sanz Penella et al. 2008).

**Table 4:** Effect of glucose and maltose on the properties of CSB

Treatment	Glucose (mg/g)	Maltose (mg/g)	Pore number	Pore area (mm^2^)	Specific volume	Hardness (kg)	Chewiness (kg)
1	0	0	1242	346.8	17.4	1600	1212
2	24.2	1.7	1566	364.7	18.3	1460	1150
3	47.4	3.0	1332	382.9	19.2	1368	1095
4	69.9	4.1	1282	416.2	19.7	1274	1007

Now, we can deduce why hardness and chewiness of CSB decreased at the FB_G_ dosage of 0.10 ml/g. Hardness and chewiness of CSB are determined by three factors i.e. protein cross-linkage, Ae/An ratio and CSB volume. Both protein cross-linkage and rise of Ae/An ratio result in the increase of hardness and chewiness, but, the larger vol_S_ decreases them. When 0.025–0.05 ml/g of FB_G_ was added to flour, the increments of hardness and chewiness from protein cross-linkage and Ae/An ratio change are more than the decrements. As a whole, hardness and chewiness of CSB are increased. When the FB_G_ dosage reaches 0.10 ml/g, the higher amylase activity catalyzes starch to produce more glucose and maltose, which results in the enlargement of vol_S_, and decrements of hardness and chewiness are more than their increments. Thus, hardness and chewiness of CSB are decreased.

In this work, FB_G_ was added to flour as a food additive and the mechanism of FB_G_ impacting on CSB properties was revealed. The FB_G_ is directly obtained from submerged cultivation, and thus its cost is cheaper than those of the purified enzymes (Victoria et al. 2013; Selinheimo et al. 2006,2007; Su et al. 2005; Shah et al. 2006). Moreover, FB_G_ containing laccase and amylases improves CSB quality significantly, the same as the purified enzymes do. It catalyzes the cross-linkage of proteins, conversion of amylopectin to amylose and degradation of starch, resulting in the changes of CSB properties including vol_S_, crumb’s micro-structure, recovery after compression, stickiness and so forth. The finding presented herein not only is helpful to further understand the functions of FB_G_ in improving CSB quality from the perspectives of protein cross-linkage and starch degradation, but also is valuable for CSB industry because it will quite likely present a relative cheap food additive. In the future investigation, except protein and starch, the effect of FB_G_ on the other components of flour also deserves to be studied. Moreover, the dosage of FB_G_ should be further studied before the extensive applications in food industry can be considered.

## Data Availability

Not applicable
